# Giardiasis in Japan, the United States of America, and Europe from 2007 to 2023, highlighting a decline during the COVID-19 pandemic

**DOI:** 10.1371/journal.pntd.0014428

**Published:** 2026-06-15

**Authors:** Hirotake Mori, Ryoko Makuuchi, Daria Krokva, Dmytro Remez, Rapeepun Prasertbun, Yoshiro Hadano, Supaluk Popruk, Aongart Mahittikorn, Rapeephan R. Maude, Dhammika Leshan Wannigama, Raita Tamaki, Gautam A. Deshpande, Roger Frutos, Chris Smith, Toshio Naito

**Affiliations:** 1 Department of General Medicine, Juntendo University Faculty of Medicine, Tokyo, Japan; 2 Department of Protozoology, Faculty of Tropical Medicine, Mahidol University, Bangkok, Thailand; 3 Division of Infection Control and Prevention, Shimane University Hospital, Izumo, Japan; 4 Division of Infectious Diseases, Department of Medicine, Faculty of Medicine, Ramathibodi Hospital, Bangkok, Thailand; 5 Department of Epidemiology, Mahidol-Oxford Tropical Medicine Research Unit (MORU), Faculty of Tropical Medicine, Mahidol University, Bangkok, Thailand; 6 Department of Infectious Diseases, Faculty of Medicine, Yamagata University and Yamagata University Hospital, Yamagata, Japan; 7 Department of Infectious Diseases and Infection Control, Yamagata Prefectural Central Hospital, Yamagata, Japan; 8 Institute of Tropical Medicine, Nagasaki University, Nagasaki, Japan; 9 School of Tropical Medicine and Global Health, Nagasaki University, Nagasaki, Japan; 10 Department of International Healthcare, Juntendo University Hospital, Tokyo, Japan; 11 Department of Internal Medicine, John A. Burns School of Medicine, University of Hawaii, Honolulu, United States of America; 12 CIRAD, URM 17, Intertryp, Montpellier, France; 13 Department of Pathology, Faculty of Medicine-Ramathibodi Hospital, Mahidol University, Bangkok, Thailand; 14 Department of Health, Faculty of Vocational Studies, Universitas Airlangga, Surabaya, Indonesia; 15 School of Public Health, Xiamen University, Xiamen, China; 16 Department of Clinical Research, Faculty of Infectious and Tropical Diseases, London School of Hygiene and Tropical Medicine, London, United Kingdom; University of Washington, UNITED STATES OF AMERICA

## Abstract

**Background:**

Giardiasis is one of the most common enteric protozoal infections worldwide. It is frequently reported as an enteric parasitic infection in the United States of America (USA) and Europe, whereas reported incidence in Japan is substantially lower. This study characterized age-specific, geographic, and temporal patterns of reported giardiasis incidence in Japan, the USA, and Europe using publicly available surveillance data.

**Methodology/principal findings:**

This retrospective study analyzed surveillance data from the National Institute of Infectious Diseases (NIID) in Japan, the Centers for Disease Control and Prevention (CDC), and the European Centre for Disease Prevention and Control (ECDC) to describe epidemiological patterns, possible explanatory factors, and temporal trends before and during the COVID-19 pandemic. Annual trends were examined using all available surveillance years: 2007–2023 for Japan and Europe and 2007–2022 for the USA. Age-specific patterns were compared between the pre-pandemic (2017–2019) and pandemic (2020–2022) periods, and geographic patterns were examined using available regional data. Incidence rates in the USA and Europe were approximately 5 per 100,000 population, whereas reported incidence in Japan remained below 0.1 per 100,000. Age-specific patterns differed: incidence peaked in children aged 0–14 years in Europe, and in children aged 0–4 years and adults aged 25–39 years in the USA, whereas in Japan, incidence was highest among adults aged 25–44 years and relatively high among those aged ≥65 years. Geographic patterns also differed, with higher incidence in Tokyo, Nordic and Baltic countries and selected European countries, and New England and Midwestern states. Annual incidence was significantly lower in the COVID-19-onset period than in the pre-COVID-19 period across all regions.

**Conclusions/significance:**

These findings suggest that exposure patterns and possible explanatory factors differ by region and age group, highlighting the importance of region- and age-specific prevention strategies.

## Author summary

Giardiasis is one of the most common protozoal infections worldwide. While it is prevalent in developing countries where sanitation is poor, it remains among the most frequently reported intestinal protozoal infections in the United States of America (USA) and Europe. Giardiasis spreads via exposure to water contaminated with *Giardia duodenalis (Giardia)* parasites, close contact with infected people or animals, and sometimes sexual contact. In Japan, however, the overall number of reported cases is much lower, and little is known about how giardiasis affects different age groups and regions compared with the USA and Europe. This study analyzed national surveillance data from Japan, the USA, and Europe from 2007 onward and compared age- and region-specific patterns before and during the COVID-19 pandemic. The results showed that giardiasis was most common in young children in Europe and the USA, whereas in Japan it mainly affected adults. Moreover, giardiasis incidence decreased in all three regions during the COVID-19 pandemic. These findings highlight regional differences in giardiasis epidemiology and may help inform future region-specific surveillance and prevention strategies.

## Introduction

Giardiasis is a parasitic infection caused by the flagellated enteric protozoan *Giardia duodenalis* (also known as *G. lamblia* and *G. intestinalis*) [1]. It is one of the most common enteric protozoal infections worldwide, affecting approximately 2% of adults and 8% of children in developed countries [1]. In contrast, Giardia infection has been reported in approximately 33% of the population in some developing countries [1]. Giardiasis may be asymptomatic or lead to a constellation of clinical symptoms including fatigue, diarrhea, nausea, abdominal cramps, bloating, weight loss, and malabsorption [1]. It has been included in the World Health Organization (WHO)’s Neglected Disease Initiative (NDI) since 2004 [2]. *Giardia* infection is primarily transmitted via the fecal-oral route through ingestion of *Giardia* cysts in human waste. Exposure can occur via consumption of water from untreated water sources such as lakes, rivers, and springs, as well as through contaminated tap water, swimming pools, or recreational water [3–7]. Previous global reviews have documented numerous waterborne outbreaks of protozoan infections worldwide, highlighting contaminated drinking and recreational water as major transmission routes and underscoring the continued public health importance of Giardia [5–7]. Contact with certain animal species and sexual activities involving fecal-oral contact have also been reported as possible risk factors in some settings [8,9]. Infections are also reported in international travelers, typically those returning from Southeast Asia, Latin America, and Sub-Saharan Africa [10].

Although giardiasis commonly occurs in developing countries due to inadequate sanitation [1], it remains among the most frequently reported enteric parasitic infections in both the United States of America (USA) and Europe [11,12]. Reported giardiasis incidence was 3.9 per 100,000 population in Europe in 2022 [11], while the reported incidence of confirmed giardiasis in the USA has remained below 7.0 per 100,000 population since 2011 [12,13]. In contrast, comprehensive national surveillance reports or peer-reviewed studies on the incidence of giardiasis in Japan remain limited. Previous studies in the USA and European countries have examined transmission routes, risk factors, surveillance patterns, and geographic variation using case-control, surveillance-based, and spatial epidemiological approaches [3,4,8,12,14–18]; however, few have comprehensively examined the broader epidemiological patterns and geographic distribution of giardiasis across Europe or the USA. To the best of our knowledge, no study has specifically compared the incidence and epidemiological differences of giardiasis among Japan, the USA, and European countries.

The COVID-19 pandemic disrupted infectious disease transmission, international travel, social contact, healthcare-seeking behavior, diagnostic testing, and public health surveillance. Declines in several gastrointestinal infections were reported during the pandemic period [19–21]. In surveillance data from Japan, CDC, and ECDC, reported giardiasis incidence also decreased in 2020, coinciding with the onset of the pandemic [13,22–24]. However, because such changes may reflect both changes in exposure and changes in healthcare-seeking or surveillance performance, their interpretation requires caution. Few studies have described age- and region-specific patterns of reported giardiasis incidence across Japan, the USA, and Europe during this period.

Using publicly available surveillance data, this study aimed to characterize age-specific, geographic, and temporal patterns of reported giardiasis incidence in Japan, the USA, and Europe, including changes observed during the COVID-19 pandemic period.

## Methods

### Data sources and case definition

This retrospective descriptive study analyzed publicly available aggregated surveillance data on giardiasis from Japan, the United States of America (USA), and Europe. These regions were selected because they have established surveillance systems that provide publicly accessible data over extended periods, allowing broad descriptive assessment of reported giardiasis incidence while recognizing differences in surveillance structures and reporting practices. Data were obtained from the National Institute of Infectious Diseases (NIID), Japan [22], the Centers for Disease Control and Prevention (CDC) [13,24], and the European Centre for Disease Prevention and Control (ECDC) [23]. Only geographic units with complete surveillance data for the relevant study period were included.

Giardiasis cases were defined according to the national or regional surveillance definitions used in each data source [13,22–24]. In general, reported cases were based on compatible clinical symptoms, such as diarrhea, abdominal discomfort, bloating, anorexia, or weight loss, together with laboratory confirmation of *Giardia duodenalis* infection. Laboratory confirmation included detection of Giardia organisms, antigens, or DNA in clinical specimens, including stool, duodenal fluid, bile, tissue samples, or small bowel biopsy specimens, depending on the surveillance system. Because this study relied on aggregated surveillance data, individual-level repeated testing, such as multiple tests in the same individual, could not be assessed. Therefore, reported cases should be interpreted as surveillance notifications rather than confirmed unique individuals. Information on co-infections with other intestinal pathogens was not available in the datasets, and asymptomatic infections were likely underreported. The geographical distribution of the study regions is shown in S1 Fig.

#### Japan.

In Japan, annual reported case counts and prefecture-level data were obtained from the National Epidemiological Surveillance of Infectious Diseases system maintained by NIID [22]. Giardiasis is classified as a Category V notifiable disease, requiring mandatory reporting of all diagnosed cases [22]. Therefore, all 47 prefectures were included in this study. Age groups were categorized as 0–4, 5–14, 15–24, 25–44, 45–64, and ≥65 years.

#### United States of America.

In the USA, annual national and state-level surveillance data were obtained from CDC surveillance summaries [13,24]. Giardiasis has been nationally notifiable since 1993, with cases reported by healthcare providers and laboratories to local or state health departments [13,24]. However, reporting requirements vary by state, and several states do not designate giardiasis as a notifiable disease. Therefore, only states with complete surveillance data were included in the analysis, resulting in a final dataset of 43 states, as shown in S1 Fig. Age groups were categorized as 0–4, 5–14, 15–24, 25–39, 40–64, and ≥65 years.

#### Europe.

In Europe, annual country-level surveillance data were obtained from the European Surveillance System (TESSy), managed by ECDC [23]. Data were included from 19 countries with complete surveillance coverage during the study period, as shown in S1 Fig. Age groups were categorized as 0–4, 5–14, 15–24, 25–44, 45–64, and ≥65 years.

### Data processing and visualization

Reported case counts and incidence rates were collected for Japan and Europe from 2007 to 2023 and for the USA from 2007 to 2022. Annual incidence trends were examined using all available surveillance years. For age-specific comparisons, the analysis was restricted to a common comparison window across the three regions: the pre-pandemic period was defined as 2017–2019, and the pandemic period was defined as 2020–2022. This restriction was applied because age-stratified data were consistently comparable across Japan, the USA, and Europe during these periods.

Mean incidence for each period was calculated using reported case counts and population data. Population data were obtained from the World Bank Open Data and the Statistics Bureau of Japan [25,26]. Because age-specific population data were not consistently available for all European countries, age-stratified incidence in Europe was calculated as the arithmetic mean of annual notification rates. The absolute reduction in incidence was defined as the difference in mean incidence between the pre-pandemic and pandemic periods.

Geographic patterns were visualized using heatmaps. Japan and Europe were summarized across four periods, 2007–2011, 2012–2015, 2016–2019, and 2020–2023, whereas the USA was summarized across two periods, 2017–2019 and 2020–2022, based on the availability of state-level data. All analyses were conducted using Microsoft Excel for Mac version 16.98 (Microsoft Corporation, Redmond, WA, USA, https://www.microsoft.com/en-us/microsoft-365/excel). Choropleth maps were generated using Datawrapper (Datawrapper GmbH, Berlin, Germany; https://www.datawrapper.de/). The map boundaries and basemap data were derived from Datawrapper’s built-in map library. Final figures were assembled and formatted in Microsoft PowerPoint. No ethical approval was required because all data used in this study were publicly available and anonymized.

### Statistical analysis

The normality of the annual incidence distributions was assessed using the Shapiro–Wilk test. For the statistical comparison shown in Table 1, all available annual incidence estimates were used. Annual incidence values from the pre-COVID-19 period, defined as 2007–2019, were compared with those from the COVID-19-onset period, defined as 2020–2023 for Japan and Europe and 2020–2022 for the USA. This comparison was descriptive and was not intended to assess the causal effect of the pandemic. Because the data were not normally distributed, the Mann–Whitney U test was used for two-group comparisons. Statistical significance was defined as p < 0.05. All statistical analyses were performed using SPSS for macOS version 29.0.2.0 (IBM Corp., Armonk, NY, USA).

**Table 1:** Comparison of annual giardiasis incidence between the pre-COVID-19 period (2007–2019) and the COVID-19-onset period (2020–2023 for Japan and Europe; 2020–2022 for the USA), using the Mann–Whitney U test.

Regions	Pre-COVID-19 period Median	COVID-19-onset period Median	p-value
Japan USA	0.05/100,000 6.06/100,000	0.03/100,000 4.44/100,000	<0.001 0.004
Europe	5.19/100,000	3.27/100,000	0.001

## Results

### Temporal trends in giardiasis incidence

**Fig 1:**
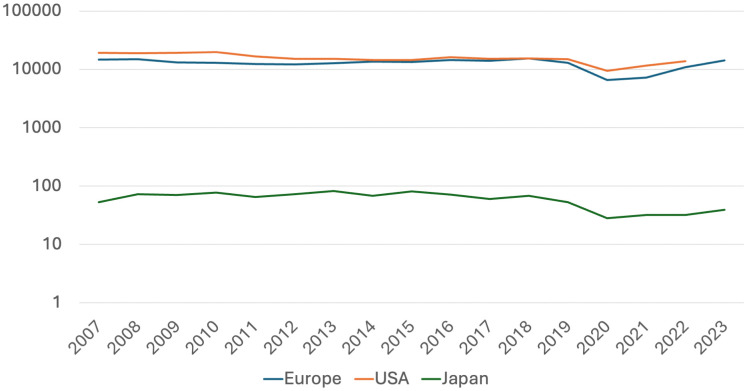
Number of giardiasis cases in Japan and Europe from 2007 to 2023 and in the USA from 2007 to 2022. The vertical axis is shown on a logarithmic scale and represents the number of reported giardiasis cases; the horizontal axis represents calendar year.

**Fig 2:**
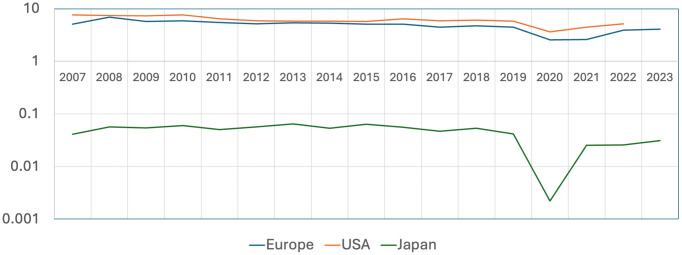
Incidence of giardiasis in Japan and Europe from 2007 to 2023 and in the USA from 2007 to 2022. The vertical axis is shown on a logarithmic scale and represents reported giardiasis incidence per 100,000 population; the horizontal axis represents calendar year.

Fig 1 shows the number of reported giardiasis cases in Japan and Europe from 2007 to 2023 and in the USA from 2007 to 2022. In most years, the USA and Europe reported over 10,000 cases annually, except during the period from 2019–2021, whereas Japan reported far fewer cases annually than the USA and Europe. Annual trends in giardiasis incidence across Japan, the USA, and Europe are shown in Fig 2. Incidence rates in the USA and Europe were generally approximately 5 per 100,000 population, whereas reported incidence in Japan remained below 0.1 per 100,000 population throughout the study period. Incidence rates were relatively stable across all regions, except between 2019 and 2020, when a marked decrease was observed. For instance, in Japan, the incidence decreased from 0.042 per 100,000 to 0.002 per 100,000, representing a 95% reduction. In contrast, the USA and Europe experienced smaller decreases during the same period, with reductions of 36.7% and 43.3%, respectively. Table 1 compares annual giardiasis incidence between the pre-COVID-19 period and the COVID-19-onset period in Japan, the USA, and Europe, showing significantly lower incidence in the COVID-19-onset period across all regions. In Japan, the median incidence dropped from 0.05 per 100,000 in the pre-COVID-19 period (2007–2019) to 0.03 per 100,000 in the COVID-19-onset period (2020–2023) (p < 0.001). In Europe, the median incidence decreased from 5.19 per 100,000 in the pre-COVID-19 period to 3.27 per 100,000 in the COVID-19-onset period (p = 0.001). In the USA, the median incidence decreased from 6.06 per 100,000 in the pre-COVID-19 period to 4.44 per 100,000 in the COVID-19-onset period (p = 0.004).

### Age-specific incidence patterns

**Fig 3:**
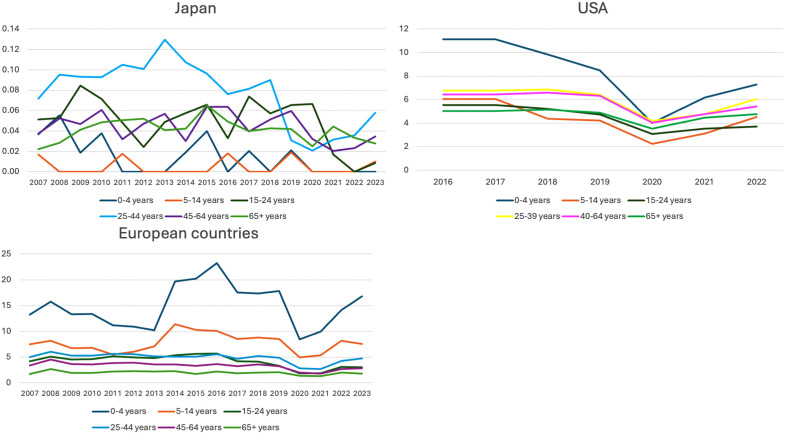
Age-specific giardiasis incidence in Japan, the USA, and European countries. Line graphs showing annual incidence per 100,000 population by age group for each region. The left vertical axes show incidence; the horizontal axes represent calendar year. Bold lines highlight key age groups illustrating distinct epidemiological patterns across regions.

The age-specific distribution of giardiasis incidence (Fig 3) reveals distinct epidemiological patterns across regions. In the USA and Europe, the highest incidence was observed in children aged 0–4 years, followed by children aged 5–14 years (in Europe), and adults aged 25–39 years (in the USA). In Europe, the incidence was lowest among older adults aged ≥65 years. In contrast, Japan exhibited a markedly different pattern, with lower incidence in children and higher incidence in young adults (15–24 years), adults (25–44 years), and individuals aged **≥**65 years. Notably, incidence among older adults increased in 2021.

### Age-specific incidence patterns across two time periods

**Fig 4:**
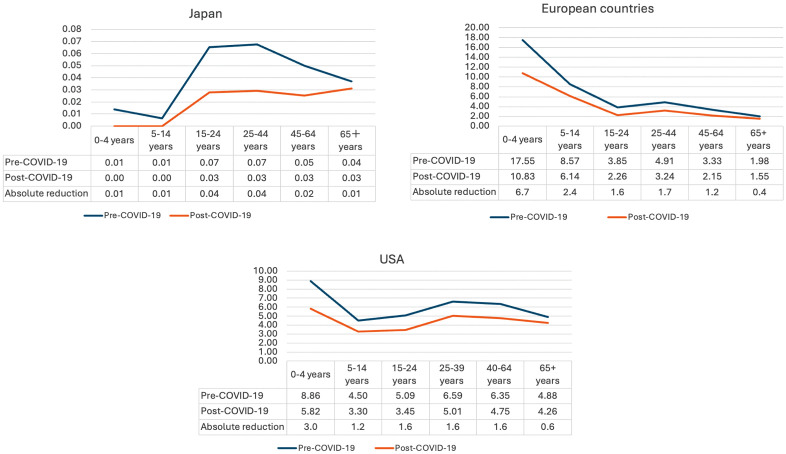
Age-specific giardiasis incidence in the pre-pandemic period (2017–2019) and pandemic period (2020–2022) in Japan, the USA, and European countries with absolute reductions for each age group. Line graphs with table showing age-specific giardiasis incidence per 100,000 population in the pre-pandemic and pandemic periods, including absolute reductions for each age group. The vertical axis shows incidence, and the horizontal axis shows age groups.

Further analysis compared age-specific mean incidence rates between the pre-pandemic period (2017–2019) and the pandemic period (2020–2022), with absolute reductions calculated for each age group (Fig 4). In Japan, the highest incidence occurred among individuals aged 25–44 years and 15–24 years in both the pre-pandemic and pandemic periods, followed by those aged 45–64 years and older. A decrease in incidence was observed across all age groups after the onset of the COVID-19 pandemic. The largest absolute reductions were observed among individuals aged 15–24 years and 25–44 years, both 0.04 per 100,000 population, followed by those aged 45–64 years, 0.02 per 100,000 population, and children aged 0–4 years, children aged 5–14 years, and adults aged ≥65 years, each 0.01 per 100,000 population. In European countries, the highest mean incidence was observed in children aged 0–4 years, followed by those aged 5–14 years and 25–44 years. Incidence decreased across all age groups in the pandemic period, with the highest absolute reductions observed in children aged 0–4 years (6.7 per 100,000 population) and 5–14 years (2.4 per 100,000 population). In the USA, the highest mean incidence was observed in children aged 0–4 years, followed by individuals aged 25–39 years. Incidence decreased across all age groups in the pandemic period, although the absolute reduction was the smallest among individuals aged **≥**65 years (0.6 per 100,000 population).

### Geographic distribution of giardiasis incidence

**Fig 5:**
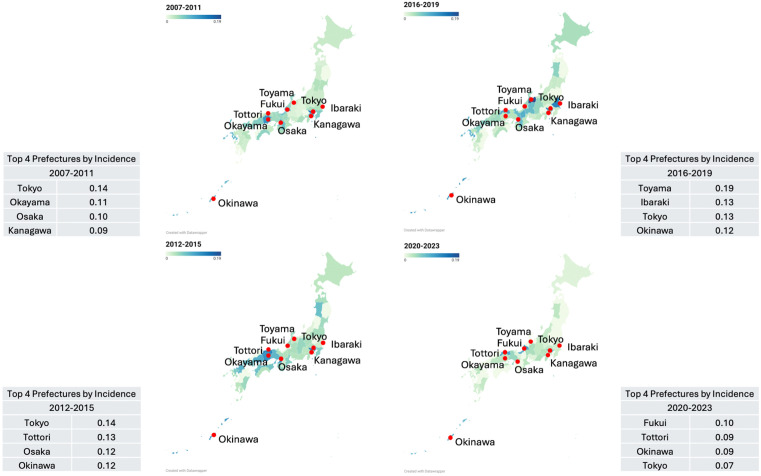
Geographic distribution of mean giardiasis incidence in Japan by prefecture across four time periods, 2007–2011, 2012–2015, 2016–2019, and 2020–2023. Heatmaps showing prefecture-level mean incidence for each period, with color gradients indicating increasing incidence. Insets list the top four prefectures by mean incidence for each period. Map boundaries were derived from Datawrapper’s built-in map library using Natural Earth Admin 1 – States and Provinces data, public domain/CC0. Natural Earth terms of use: https://www.naturalearthdata.com/about/terms-of-use/. Created with Datawrapper.

**Fig 6:**
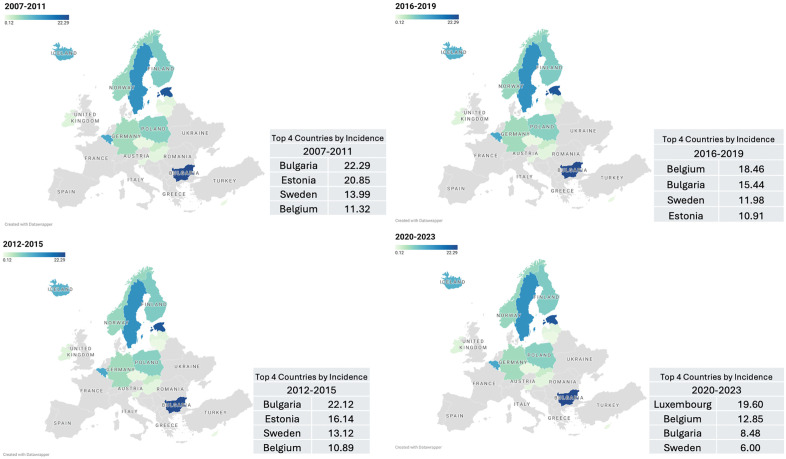
Geographic distribution of mean giardiasis incidence in Europe by country across four time periods, 2007–2011, 2012–2015, 2016–2019, and 2020–2023. Heatmaps showing country-level mean incidence for each period with color gradients indicating increasing incidence. Insets list the top four countries by mean incidence for each period. Map boundaries were derived from Datawrapper’s built-in map library using Natural Earth Admin 0 – Countries data, public domain/CC0. Natural Earth terms of use: https://www.naturalearthdata.com/about/terms-of-use/. Created with Datawrapper.

**Fig 7:**
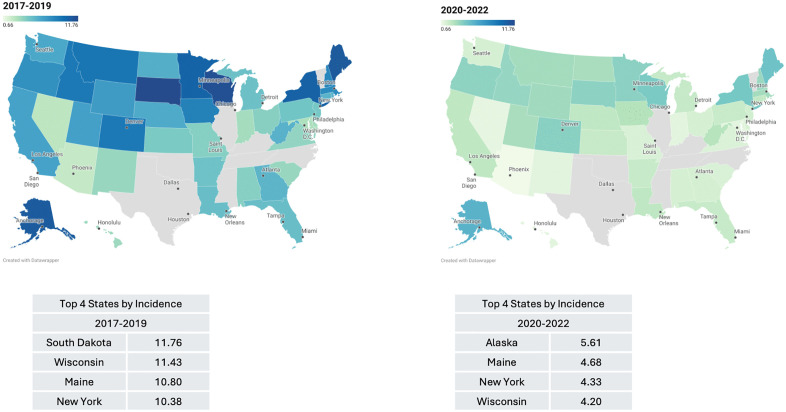
Geographic distribution of mean giardiasis incidence in the USA by state across two time periods, 2017–2019 and 2020–2022. Heatmaps showing state-level mean incidence for each period, with color gradients indicating increasing incidence. Insets list the top four states by mean incidence for each period. Map boundaries were derived from Datawrapper’s built-in map library using **U.**S. Census Bureau boundary data, public domain/CC0. **U.**S. Census Bureau TIGER/Line Shapefiles: https://www.census.gov/geographies/mapping-files/time-series/geo/tiger-line-file.html. Created with Datawrapper.

Geographic variation in giardiasis incidence across different time periods is shown in Figs 5–7. In Japan and European countries, the observation period was divided into four periods: 2007–2011, 2012–2015, 2016–2019, and 2020–2023. In the USA, the observation period was divided into two periods: 2017–2019 and 2020–2022. In Japan, mean incidence across four periods (2007–2011, 2012–2015, 2016–2019, and 2020–2023) showed that the highest-incidence prefectures were Tokyo, Okayama, Osaka, and Kanagawa (2007–2011); Tokyo, Tottori, Osaka, and Okinawa (2012–2015); Toyama, Ibaraki, Tokyo, and Okinawa (2016–2019); and Fukui, Tottori, Okinawa, and Tokyo (2020–2023). In European countries, the highest-incidence countries were Bulgaria, Estonia, Sweden, and Belgium (2007–2011 and 2012–2015); Belgium, Bulgaria, Sweden, and Estonia (2016–2019); and Luxembourg, Belgium, Bulgaria, and Sweden (2020–2023). In the USA, the states with the highest incidence were South Dakota, Wisconsin, Maine, and New York (2017–2019); and Alaska, Maine, New York, and Wisconsin (2020–2022).

## Discussion

This study identified clear differences in giardiasis incidence across Japan, the USA, and Europe, together with distinct age-specific, geographic, and temporal patterns, including a marked decline in incidence beginning in 2020 across multiple regions (Table 1).

Compared with the USA and European countries, Japan exhibited a lower number of cases and overall incidence, with a distinct age-specific distribution showing a higher incidence among adults aged 25–44 years, in contrast to the predominance of cases in children under five years of age in the USA and European countries (Fig 3). This epidemiological divergence likely reflects multiple factors, including differences in age-related behaviors and exposure [3,4,16,27,28], regional variation in the role of waterborne transmission [5–7,29] and sewage sludge management [30–38], and the influence of international travel and migration [10,39–41]. In the USA and Europe, young children are more vulnerable due to common exposure to contaminated recreational water and close contact in childcare settings [16,27,28,42]. Previous studies have identified key risk factors such as drinking untreated water, swimming in rivers, lakes, or streams, and contact with diapered children in the USA [16,27,28]. In Europe, risk factors include exposure to swimming pools (e.g., Belgium), agricultural and recreational water (e.g., France), and consumption of large volumes of drinking water or contact with agricultural water (e.g., Italy) [14,16,42]. In contrast, the predominance of giardiasis cases among adults in Japan likely reflects behavioral and social risk factors specific to this age group, notably international travel, especially to high-prevalence regions such as India, and transmission among men who have sex with men (MSM) [43]. These adult-specific exposures may partly explain the unique age distribution observed in Japan.

Waterborne transmission remains a major risk factor in the USA and European countries, as consistently highlighted in previous global reviews [5–7]. During 2012–2017, officials from 26 US states reported 111 giardiasis outbreaks (760 cases) to the National Outbreak Reporting System (NORS), of which 26% were linked to water exposure [29]. In Europe, although no national or multi-country outbreaks were reported in 2019 or 2022, a large outbreak occurred in Italy during 2018–2019, attributed to tap water [11,44]. In contrast, Japan reported only four waterborne outbreaks among 578 cases between 2006 and 2013 [43], suggesting the lesser significance of this route. This likely reflects Japan’s comprehensive water management system, characterized by centralized regulation, nationwide monitoring, residual chlorine standards (>0.1 mg/L), strict watershed protection, together with lower reliance on private wells and reduced exposure to untreated water compared with the USA and Europe [7,45].

In addition, differences in sewage sludge management may also contribute to these contrasting risks. In the USA and many European countries, a substantial proportion of sewage sludge is reused in agriculture, exceeding 50% in Bulgaria and Sweden [30–32,46]. Such practices may raise concerns regarding microbial contamination, as sewage sludge often contains *Giardia* cysts and other pathogens, including *Escherichia coli* and other intestinal bacteria [33]. Although *Giardia* cysts have been detected in sewage sludge [33,34], quantitative microbial risk assessments (QMRA) indicate that infection risk is extremely low (10 ⁻ ⁷–10 ⁻ ⁴) [35,36]. This aligns with the WHO health-based target (HBT) (e.g., 10 ⁻ ⁶ per person-year), which emphasizes exposure reduction rather than absolute pathogen elimination [37]. European and U.S. systems apply multiple barriers, such as harvest intervals, setback distances, and access control [31,36,46,47].

Conversely, Japan employs thermal treatment for nearly all sewage sludge, in which incineration or carbonization processes at 800–900 °C are used for both volume reduction and energy recovery [38]. The widespread adoption of thermal treatment in Japan may reduce the environmental circulation of enteric pathogens and could partly contribute to the low incidence observed in Japan. Therefore, although QMRA-based evaluations in Europe and the United States suggest that sludge-associated infection risk is acceptably low under HBT approaches, the markedly lower giardiasis incidence observed in Japan raises the possibility that differences in sludge treatment may further contribute to reducing transmission.

Migration is another factor contributing to the observed epidemiological differences. Studies in Europe and the USA have shown disproportionately higher giardiasis incidence among migrants from endemic regions [39,40]. Such cases linked to travel or socioeconomic and living conditions may partly explain the higher incidence in these regions. For instance, in Miami, 62% of Giardia-positive laboratory results between 2011 and 2014 occurred in Hispanics, many with travel ties to Latin America [41]. Similarly, Swedish national data reported a giardiasis risk of 1,180 per 100,000 among immigrants and refugees [40]. Although direct evidence is limited, these findings suggest that imported infections among immigrants may contribute to the higher incidence in Europe and the USA than in Japan, where the migrant population is smaller. Further research is needed to clarify this contribution.

Another notable age-specific pattern was observed among adults aged ≥65 years. In Japan, incidence in this age group was relatively high, whereas a similar but less pronounced pattern was observed in the USA; in contrast, incidence among adults aged ≥65 years remained low in Europe (Fig 3). Several factors may contribute to this difference, although these explanations remain speculative in the absence of individual-level exposure data. These include age-related changes in susceptibility, Japan’s rapidly aging population structure, and possible environmental exposures related to elderly care services. For example, day-care services for older adults and home-based bathing assistance could represent potential opportunities for transmission. Further studies are needed to better understand why the incidence among older adults in Europe is substantially lower than in Japan and the USA.

The geographic distribution of giardiasis showed distinct region-specific patterns. In Japan, Tokyo was consistently among the highest-incidence, which may reflect its large young adult population, greater international travel, and more frequent social contact (Fig 5). By contrast, a similar urban-centered pattern was not observed in Europe or the USA. In Europe, incidence was relatively high in Nordic and Baltic countries such as Sweden and Estonia, as well as in Bulgaria and Belgium (Fig 6). These geographic patterns may partly reflect environmental conditions, although direct evidence linking climate and giardiasis incidence in these settings remains limited [48,49]. In the USA, incidence was higher in New England and the Midwest, where reliance on private wells is greater (Fig 7) [17,18,24].

During the COVID-19 pandemic, the overall incidence of giardiasis declined across Japan, the United States, and Europe (Fig 2 and Table 1). This decline may reflect both reduced opportunities for transmission and changes in surveillance performance. Public health measures, including social distancing, improved hand hygiene, travel restrictions, and home isolation, may have reduced exposure to gastrointestinal pathogens, while reductions in case detection, recording, and reporting within public health surveillance systems may also have contributed to the observed decline [19,50]. The magnitude of decline differed by age group and region (Fig 4). In Japan, the largest reduction was observed among adults, possibly reflecting the impact of international travel restrictions, whereas in the USA and Europe, the largest reductions were observed among children, possibly reflecting reduced exposure in childcare settings, recreational water, and natural water sources. Although few previous studies have examined age-specific declines in giardiasis incidence, our findings suggest that pandemic-related behavioral changes may have had the greatest effect on the age groups with the highest baseline exposure risk in each region. In contrast, the geographic pattern of high-incidence areas remained broadly similar before and during the pandemic. In the USA and Europe, high-incidence areas were broadly similar across periods, whereas in Japan, several prefectures, including Tokyo and Okinawa, repeatedly appeared among the higher-incidence areas. (Figs 5–7), suggesting that pandemic-related restrictions may have reduced overall transmission without substantially altering the underlying regional risk structure.

This study was based on aggregated surveillance data and therefore represents a descriptive epidemiological analysis rather than an investigation of transmission mechanisms or individual-level risk factors. Accordingly, some limitations should be considered, including the inability to examine routes of transmission or statistically assess risk factors within each region due to the lack of individual-level data. Because the analysis used aggregated surveillance data, repeated testing, duplicate notifications, co-infections, and asymptomatic infections could not be fully assessed. In addition, interpretation of the geographic distribution presented in the US heatmap should be made cautiously, as surveillance systems and reporting practices differ across states. More broadly, differences in surveillance systems, diagnostic practices, healthcare-seeking behavior, and reporting requirements across regions may have influenced the observed differences in reported incidence. Therefore, the findings should be interpreted as differences in reported surveillance incidence rather than true infection incidence.

In conclusion, Japan has a notably lower incidence of giardiasis than the USA and European countries, with the incidence peaking in adults rather than young children. These differences may reflect robust water infrastructure, unique sewage sludge management, limited recreational exposure to untreated water, a predominant link with international travel, and a relatively small migrant population. In contrast, waterborne transmission remains a key factor in the USA and Europe, particularly among children. Across all three regions, giardiasis incidence decreased during the COVID-19 pandemic, most notably in groups with the highest pre-pandemic risk, suggesting that pandemic-related behavioral and surveillance changes may have influenced observed incidence patterns. These findings highlight regional differences in giardiasis epidemiology and underscore the importance of continued surveillance and region- and age-specific prevention strategies.

## Supporting information

S1 Fig Geographic distribution of study regions included in the analysis. The figure shows Japanese prefectures, US states, and European countries included in the analysis. Map boundaries were derived from Datawrapper’s built-in map library using Natural Earth Admin 0 – Countries, Natural Earth Admin 1 – States and Provinces, and U.S. Census Bureau boundary data, public domain/CC0. Natural Earth terms of use: https://www.naturalearthdata.com/about/terms-of-use/; U.S. Census Bureau TIGER/Line Shapefiles: https://www.census.gov/geographies/mapping-files/time-series/geo/tiger-line-file.html. Created with Datawrapper.
(TIF)
